# Genomic insights from a long-read assembly of a deep-sea *Chromohalobacter israelensis* strain from Santos Basin pockmarks field

**DOI:** 10.1007/s42770-026-02057-w

**Published:** 2026-08-19

**Authors:** Ian Ariel Barbosa Nunes, Adan Rodrigues de Oliveira, Adonney Allan de Oliveira Veras, Artur Silva, Elielson Cabral Costa, Flúvio Modolon, Francielli Vilela Peres, Michel Michaelovitch de Mahiques, Rafael Azevedo Baraúna, Sávio de Souza Costa, Diego Assis das Graças, Vivian Helena Pellizari

**Affiliations:** 1 Instituto de Ciências Biológicas, Universidade Federal do Pará, Pará, Brazil; 2 Instituto Oceanográfico, Universidade de São Paulo, São Paulo, Brazil; 3 Laboratório de Engenharia Biológica - EngBio, Parque de Ciência e Tecnologia do Guamá, Espaço Inovação, 2° andar, Universidade Federal do Pará, Rua Augusto Corrêa, 01, Pará 66075-110 Belém, Brazil

**Keywords:** Halophiles, Genome, Pockmarks, Salt diapirs

## Abstract

Continental slopes, particularly the pockmark and salt diapir regions of the Santos Basin, represent extreme environments characterized by high hydrostatic pressure, low temperatures, elevated salinity, and limited organic matter, fostering unique bacterial communities. This study aimed to elucidate the metabolic strategies enabling survival in these conditions by exploring the genome of a *Chromohalobacter israelensis* strain isolated from such sediments, utilizing long-read sequencing. The genome, assembled into a single 3.8 Mb contig with 98.9% completeness, confirmed the strain’s taxonomic identity. Analysis revealed genes for metabolizing diverse carbohydrate sources, a complete ectoine synthesis cluster essential for halophily, and genes conferring tolerance to osmotic stress, low temperatures, and pH, underscoring its polyextremophilic capacity. Pangenome analysis identified a substantial core genome with essential metabolic functions, including a species-exclusive sulfur metabolism reaction. These findings highlight the strain’s biotechnological potential and contribute to understanding the genus’s adaptation to diverse hypersaline habitats and the strain’s potential role in the sampled environment.

**Supplementary Information:**

The online version contains supplementary material available at 10.1007/s42770-026-02057-w.

## Introduction

Continental margins, while constituting only 20% of the oceanic area, account for up to 50% of marine productivity [1, 2]. Although exposed to various extremes, the deep environments of continental margins converge with the rest of the deep sea, representing the planet’s largest habitat and harboring high genetic and microbial diversity [3, 4]. Knowledge regarding these communities remains scarce, particularly in the South Atlantic [5]. Nevertheless, it is known that they play essential roles in seafloor environments, participating in biogeochemical cycles, organic matter remineralization, and primary production [6].

The sampling sites for this study, located in a slope region of the Santos Basin in São Paulo, present unique conditions due to the presence of a pockmark and salt diapir field [7]. Pockmarks are concave depressions formed after the rapid release of fluids trapped beneath the sediment [8], influencing the ecosystem by affecting water flow, the type and quantity of available food, and sedimentation rates [9]. Furthermore, they can play a significant role in structuring benthic communities and acting as biodiversity refuges, as they exhibit greater diversity than the surrounding sediment [10]. Salt diapirs, on the other hand, are large salt reservoirs that, when exposed to the seafloor surface and interacting with local bottom currents, render the sediment excessively saline [11]. Additionally, slopes often exhibit high sediment accumulation [7], with the Santos Basin pockmarks, in particular, considered natural sediment traps [12], which characterizes them as selective sites for halophilic microorganisms.

Understanding the genetic potential of organisms in marine sediment environments is crucial for elucidating their metabolic capabilities and predicting their responses to environmental disturbances [13, 14]. Among these microorganisms, the bacterium *Chromohalobacter israelensis*, belonging to the family *Halomonadaceae*, stands out. The bacterium grows in media with 0.9% to 25% salt content, from 5 to 10 pH and from 15 to 45 °C. Optimal growth occurs in 7.5 to 10% salt concentration, pH 7.5 and 37 °C [15]. This species is a model organism for osmoregulation studies due to its ability to survive using various substances as a sole carbon source and its affinity for hypersaline environments [16]. Although its genomes have been previously published [17], there are no recorded instances of this species in pockmark regions in the literature. The species employs compatible solute accumulation strategies to protect itself from extreme environmental stresses [18], producing multifunctional stabilizing molecules of significant commercial interest [19].

This study presents a long-read genomic analysis, utilizing nanopore sequencing, of an undeposited strain of *Chromohalobacter israelensis* (herein referred to as SB 259). The objectives were to complete the genome of this strain, elucidate the genomic mechanisms responsible for the taxon’s vital functions through genomic annotation and pangenome analysis, identify genes that may provide adaptations to extreme environments and reinforce its taxonomy. A complete genome was obtained and annotation revealed diverse stress tolerance genes, the pangenome holds a robust core genome where most mobile genetic elements from the mobilome can be found, suggesting an evolutionary strategy that would enable the genus presence in the diverse habitats it can be found. A complete ectoine synthesis gene cluster was found, highlighting the biotechnological potential of the strain.

## Methods

### Isolation

The strain characterized in this study was previously isolated from marine sediments collected at a depth of 559 m from a salt diapir (station 259) located within a pockmark field in the Santos Basin, Southwest Atlantic Ocean, as described by Peres et al. [20].

Initially, the culture was enriched by adding 2 g of sediment to 50 mL flasks containing hypersaline culture medium (25% NaCl) (HM medium - Halobacterium) [21]. This was then incubated aerobically at 20 °C for two months. The enriched cultures were subsequently transferred to liquid media with 25% and 10% NaCl to optimize the recovery of halophilic microorganisms. Following growth, the enriched cultures were transferred to solid media at the same saline concentrations, where unique colonies were isolated to obtain pure cultures. The isolates had their 16s rRNA gene amplified and sequenced for identification.

### DNA extraction, sequencing and assembly

DNA extraction from the isolates was performed using the Quick-DNA Fungal/Bacterial Isolation kit (Zymo Research, USA). Purity analysis was conducted on a NanoDrop spectrophotometer (Thermo Fisher Scientific), and quantification was done with the Qubit dsDNA HS kit (Thermo Fisher Scientific, São Paulo, Brazil). Following extraction and quantification, the DNA of isolate SB259 was provided by Peres et al. Sequencing was executed on the PromethION 2 Solo platform (Oxford Nanopore Technologies) utilizing the R10.4.1 flowcell with the Rapid Sequencing DNA V14 kit and native barcoding kit 96V14 following the manufacturer’s instructions for library preparation, at the Laboratory of Biological Engineering in Belém, Pará, Brazil.

Raw data were processed with NanoPlot v1.42.0 [22] and Filtlong v0.2.1 [23] for raw reads analysis and filtering keeping reads with at least 790 bases of length and the 90% largest reads, followed by de novo assembly using NextDenovo v2.5.2 [24] via an automation script (available at https://github.com/ian-ariel/AutoAssembly). Assembly quality was assessed with Quast v5.3.0 [25], completeness with Busco v5.8.3 [26] and CheckM2 [27], which also calculated contamination. Completeness and contamination were also calculated using the reference genome for means of comparison. The assembly was subsequently polished by the software ContigPolishing [28] without using short reads. Circularization was tested by running Circlator v1.5.5 [29] with the output of Dnaapler v1.3.0 [30].

### Genome analysis

For genomic analysis, Average Nucleotide Identity (ANI) was calculated using FastANI v1.34 [31] against the eight *Chromohalobacter* reference genomes available in the NCBI RefSeq database (accession numbers: *C. israelensis* ASM5578v1; *C. canadensis* ASM3447955v1; *C. japonicus* ASM2306117v1; *C. moromii* ASM2309186v1; *C. marimortui* ASM436431v1; *C. sarecensis* ASM2306113v1; *C. beijerinckii* ASM2306118v1; *C. nigrandensis* ASM2306128v1) to reinforce the strain’s taxonomic identification. Digital DNA-DNA Hybridization (dDDH) was determined via the Genome-to-Genome Distance Calculator (TYGS) [32] with the same purpose. The detection of resistance genes, virulence genes, and plasmids was performed using ResFinder 4.7.2 [33], VirulenceFinder 2.0 [34], and PlasmidFinder 2.1 [35], respectively.

For functional genomic annotation, multiple approaches were employed separately to identify important functions present in the genomes and have it confirmed by different tools: PGAP v6.10 [36] (as the main annotation to be used in tools requiring annotation formatted files). To cross-validate general metabolic predictions and resolve potential annotation discrepancies, the genome was parallel-processed using RAST-tk v1.9.5 [37], Prokka v1.14.5 [38], blast-KOALA v3.1 [39] and PATRIC [40]. Specialized tools were used to target non-overlapping ecological traits: DRAM v1.5.0 [41] was utilized to distill carbohydrate-active enzymes (CAZymes) and complex carbon metabolisms; METABOLIC v4.0 [42] was utilized to scale annotations into community-level biogeochemical cycling profiles. Biosynthetic gene clusters were identified using antiSMASH v8.0 [43]. The R package “microTrait” v1.0.0 [44] was utilized to extract fitness traits from the genomic sequences. Pangenome analysis was conducted with Anvi’o v8 [45], incorporating the reference genomes of the genus and the assembled genome, following the anvi-pan-genome script. The stress tolerance genes annotated were subsequently compared to the strain’s core genome, previously defined through pangenome analysis, to verify the distribution and prevalence of stress tolerance traits within the core region.

Mobilome characterization was performed by identifying and evaluating various mobile genetic elements (MGEs), including CRISPR-Cas systems, bacteriophages, genomic islands, insertion sequences, and transposable elements. The following bioinformatics tools were employed for this purpose: CRISPRCasFinder [46] for detecting CRISPR-Cas systems, IslandViewer 4 [47] for predicting genomic islands, and ISfinder [48] for insertion sequences, considering predictions with identity values less than 1 × 10^− 5^ and excluding hits with more than 1^− 10^ e. value. Additionally, analysis generated by AlienHunter [49] within the score threshold of 10.408 and keeping regions above 5 kb of length, VirSorter [50], and Phigaro [51] were performed using the ProkSee platform [52].

### Phylogenomics

A phylogenomic dataset was constructed using 1,512 orthologous groups, corresponding to 15,120 (across the 10 genomes included in the pangenome) single-copy core genes (SCGs), which were retrieved and concatenated via the anvi-get-sequences-for-gene-clusters tool from the Anvi’o platform. The obtained sequences were aligned using MUSCLE v5.1 [53], followed by refinement with trimAl [54] (parameter -gt 0.50) to remove alignment columns with gap occupancy exceeding 50%. Model evaluation was performed using the automatic model evaluation framework in RAxML-ng v1.2.2 [55], which identified the LG matrix with optimized empirical amino acid frequencies and a mean gamma distribution across 8 categories (LG + FC+G8m) as the optimal evolutionary model. Phylogenetic inference was performed using the maximum likelihood (ML) approach under this optimized model. Ten randomized parsimony trees were generated as starting points to mitigate topological bias. Node support was assessed via 1,000 non-parametric bootstrap replicates. The final topology was visualized and annotated in iTOL v7 [56].

### Data and code availability

For verifiability of this work, the scripts were deposited in the following git https://github.com/ian-ariel/chromohalobacter .

## Results

### Sequencing, assembly and taxonomic delineation

Sequencing yielded 992.4 Mb of long reads in 245,893 reads with a mean N50 of 3,544 bases, resulting in a complete genome of 3,809,067 bp in a single contig, with a GC content of 63.78% (Table 1). Before filtering 99.1% of the reads presented Q > 10 and 61.9% presented Q > 15. After filtering, the Q > 10 value was 100% and the Q > 15 was 74.5%. The filtering step removed 43% of the reads, improved the mean read length to 3,034.0 (1,903.6 before filtering) and the mean N50 to 3,948 bases. The effective coverage used for assembly was estimated at 110.59x based on the final genome assembled. ContigPolishing took two rounds of refining to return the final assembly. The assembly demonstrated 98.9% completeness regarding *Halomonadaceae* family marker genes (total of 973 marker genes evaluated) on BUSCOs database halomonadaceae_odb12; which also estimated 0% duplicated marker genes, 0.7% fragmented markers and 0.4% missing markers (99.9% completeness and 0.1% fragmented markers in the reference genome). The CheckM2 analysis returned 100% completeness and 0.33% contamination (100% completeness and 0.04% contamination in the reference genome). The results from the previous softwares may be overestimated by the very closely related references. Circlator returned a circularized genome from the reoriented Dnaapler output. ANI analysis revealed 98.023% similarity to *Chromohalobacter israelensis*, surpassing the 96% threshold for species identification [57]. The dDDH values were 82.5% with *C. israelensis 6768* and 75.5% with *C. israelensis* DSM 3043 (Table 2), both above the 70% threshold for species delimitation [58].

**Table 1:** Assembly properties of SB 259 and pre-polishing assembly, including contig statistics genome size, GC content and predicted gene count

Assembly properties	SB 259	Pre-polishing
Contigs	1	2
Total length (bp)	3,809,067	3,821,467
GC (%)	63.78	63.75
Predicted genes	3595	-
Number of features (RAST)	3658	-
Number of subsystems (RAST)	308	-
Number of RNAs (PGAP)	88	-

**Table 2:** Taxonomy statistics including ANI and dDDH values for SB 259 against the genus *Chromohalobacter*

Reference Genome	SB 259		
	ANI (%)	dDDH(%)	Genome length (bp)
*C. israelensis DSM 3043*	98.02	75.5	3 696 649
*C. marismortui*	86.92	61.9	3 553 220
*C. moromii*	85.91	63.2	3 470 002
*C. beijerinckii*	85.71	64.1	3 340 801
*C. canadensis*	85.66	62.6	3 595 165
*C. japonicus*	85.62	60.8	3 436 593
*C. sarecensis*	83.93	53.8	3 490 490
*C. nigradesensis*	83.73	49.3	3 636 986

### Structural and functional genome analysis

**Fig. 1:**
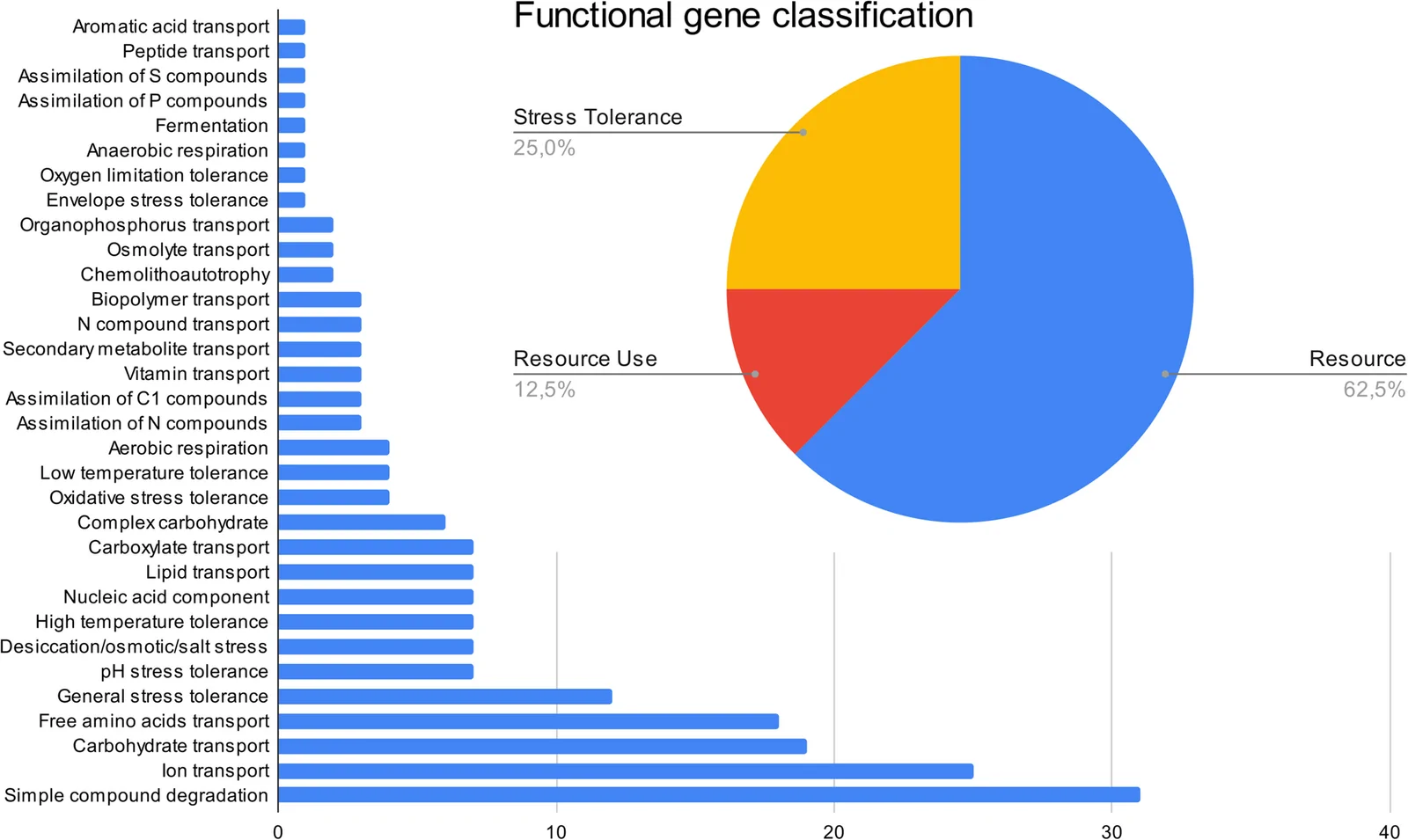
Annotation-Based Comparative Analysis of the *C. israelensis* genome. The figure displays the functional gene classification within the SB 259 genome, based on microtrait annotation

Tools like PlasmidFinder, ResFinder, and VirulenceFinder did not detect plasmids, antibiotic resistance genes, or virulence genes. Functional annotation by the MicroTrait v1.0.0 software revealed a highly diverse metabolism, classifying 59.75% of genes as participants in resource acquisition processes: protein degradation, transport of free amino acids, transport of metal and inorganic ions, monosaccharides, polysaccharides, and lipids. Additionally, 21.6% were classified as stress tolerance genes, and 18.9% as resource utilization genes (Fig. 1).

**Fig. 2:**
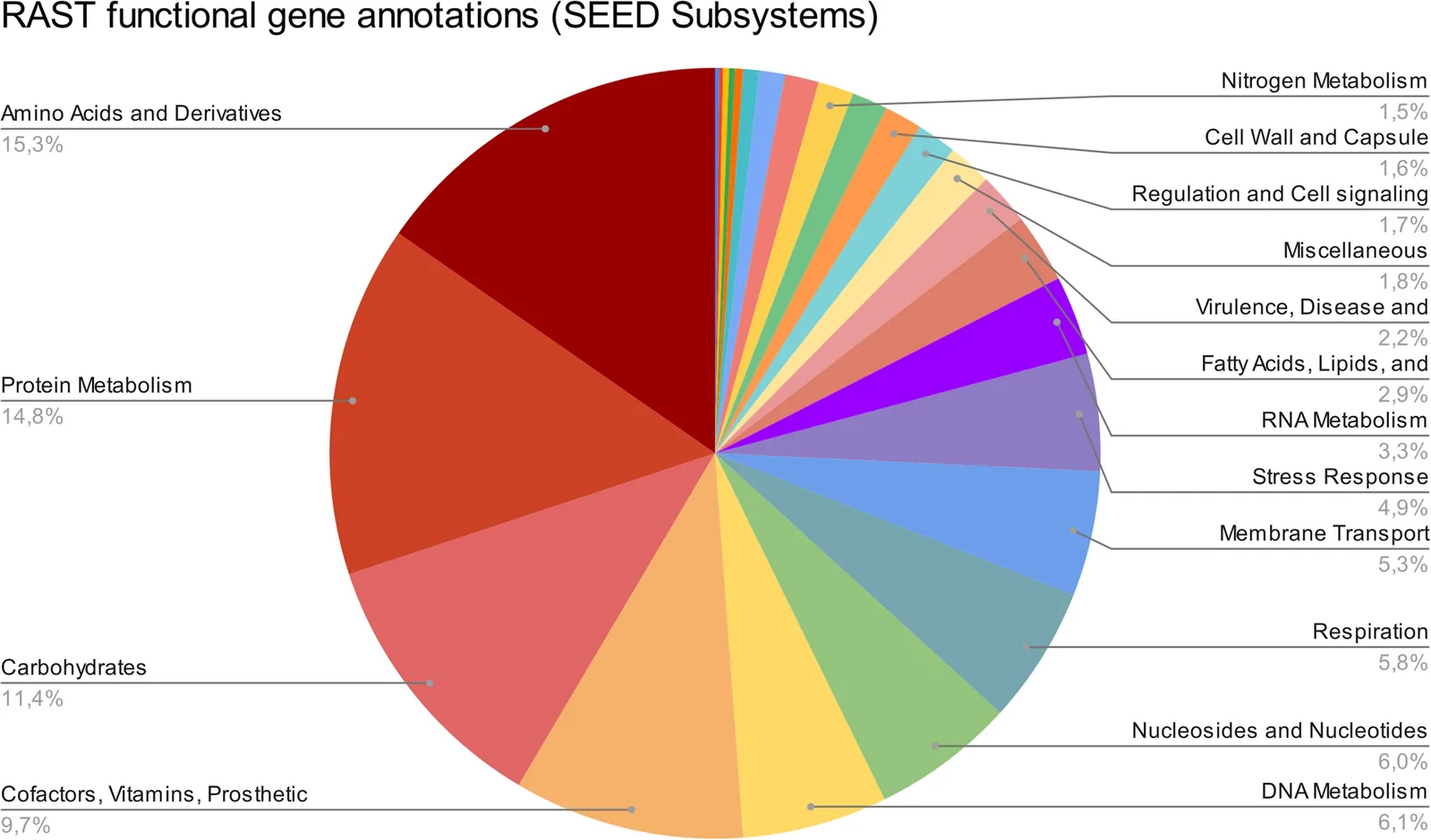
Comparative analysis of *C. israelensis* genome annotation according to RAST

RAST identified 3,575 genes, 308 metabolic subsystems, and 83 RNAs (Fig. 2). The functional annotation from the PGAP v6.10 software identified 3,575 genes and 88 RNAs.

The assessed mobilome revealed 186 hits for transposase in the Prokka software annotation, 3 hits for CRISPR sequences, and 1 hit for a Cas enzyme cluster on the CRISPRCasFinder platform (Online Resources S6 and S7). The IslandViewer4 platform predicted 29 genomic islands (Online Resource S8), while 73 sequences yielded significant alignments against the ISFinder database (Online Resource S9). Additionally, the ProkSee platform generated results (Online Resources S10-S12) from the following tools: Phigaro detected 2 prophage sequences; VirSorter found 1 viral sequence; and AlienHunter identified 50 putative horizontal gene transfer (HGT) events.

### Metabolism and adaptation mechanisms

The energy metabolism includes genes for nitrogen cycling (nitrate/nitrite ammonification, urease. e.g., nit-6, narG, narH, nasC), sulfur cycling (sulfide reduction/oxidation, sulfate. e.g., TrxR, cysJ, cysN, cysD), and phosphorus cycling (Polyphosphate kinase EC 2.7.4.1) (Table 3). Stress tolerance genes were identified for high temperatures (Hsp20), pH (e.g., COG1220, COG5405), oxidative stresses (e.g., PerR, SODFe/Mn), and, mainly, hypersalinity (e.g., BetA, CodA). Genes for the biosynthesis (ectABCD) and degradation (doeABC) of the compatible solute ectoine were annotated, as were those for the ectoine tripartite ATP-independent periplasmic (TRAP) transport system (teaABC) and for betaine biosynthesis from choline dehydrogenase. Genes for arsenic/mercury detoxification (e.g., arsABC, arsR1, merATR, merR1) and motility systems (flagellum and chemotaxis e.g., flhA_1, flhA_2, fliD_1, fliD_2, fliS, fliT, cheA, cheW, cheR) were also highlighted. Finally, broad homology-based annotations from RAST and DRAM flagged putative antimicrobial resistance genes (e.g., oqxB12); however, because ResFinder, a tool that specifically targets horizontally acquired resistance genes, yielded negative results, these RAST and DRAM hits likely represent intrinsic (non-mobile) resistance mechanisms or false-positive functional assignments due to sequence similarities.

The stress tolerance genes (Online Resource S1) mapped against the core genome coordinates revealed that 41 genes were within the core genome coordinates (Online Resource S2), including the genes for ectoine synthesis, degradation and transport.

**Table 3:** Ecologically relevant functions associated with *C. israelensis* SB 259, including carbohydrate utilization, phosphorus metabolism, nitrogen metabolism, sulfur metabolism, glycoside hydrolases, and other metabolic functions

Characteristic	Presence	Characteristic	Presence
Carbohydrate utilization and degradation		Carbohydrate active enzymes	
Fructose ᵃ, ᵉ	“+”	Amidase ᵉ, ᵍ, ʰ	“+”
Galactose ᵉ	“+”	Beta-xylosidase ᵉ, ᵍ, ʰ	“+”
Mannose ᵇ	“+”	Beta-glucosidase ᵉ	“+”
Xylose ᵃ, ᵇ, ᵉ	“+”	Alpha-glucosidase ᵉ	“+”
Lignin ᵉ	“+”	Beta-N-acetylglucosaminidase ᵉ, ʰ	“+”
Cellulose ᵃ	“+”	Pyruvate-kinase ᵉ, ᵍ, ʰ	“+”
Chitin ᵃ, ᵉ	“+”	Pullulanase ᵉ	“+”
C1 assimilation by methanotrophy ᵃ	“+”	**Stress tolerance**	
Xylan ᵃ, ᵉ	“+”	Low temperature ᵃ	“+”
Arabinan ᵉ	“+”	pH ᵃ	“+”
Starch ᵉ	“+”	Oxidative ᵃ, ᵇ	“+”
Mucin ᵉ	“+”	Oxygen limitation ᵃ	“+”
Pectin ᵉ	“+”	High temperature ᵃ	“+”
**Phosphorus metabolism**		Salt/Desiccation/Osmotic ᵃ, ᵇ, ᶠ	“+”
Phosphatase ᵇ, ᵍ, ʰ	“+”	**Other metabolic functions**	
Alkaline -phosphatase ᵃ, ʰ	“+”	Phosphoenolpyruvate -carboxylase ᵇ, ᵍ, ʰ	“+”
**Nitrogen metabolism**		Iron acquisition ᵇ, ᵈ, ᵍ, ʰ	“+”
Ammonia/ammonium assimilation ᵃ, ᵇ, ᵉ, ᵍ	“+”	Iron -reduction ᶜ	“+”
Nitrate and nitrite ammonification ᵇ, ᵈ, ᵉ, ᵍ, ʰ	“+”	Flagellum ᵇ, ᵈ, ᵉ, ᵍ, ʰ	“+”
Nitrite -oxidation ᵃ, ᵈ, ᵉ	“+”	Chemotaxis ᵈ, ᵍ, ʰ	“+”
Urease (ureABC) ᵃ, ᵍ, ʰ	“+”	Antimicrobial resistance ᵇ, ᵉ, ᵍ, ʰ	“+”
**Sulfur metabolism**		Arsenic reduction ᵈ, ᵉ	“+”
Sulfide reductase ᵇ, ᵍ, ʰ	“+”	Mercury reduction ᵈ, ᵉ, ᵍ, ʰ	“+”
Sulfate reduction ᵃ, ᵉ, ʰ	“+”	Catalase ᵍ, ʰ	“+”
Sulfur -assimilation ᵈ, ʰ	“+”		
Sulfide oxidation ᵃ, ᵈ, ʰ	“+”		

Microtrait - ᵃ; RAST - ᵇ; METABOLIC - ᶜ; KEGG Decoder - ᵈ; DRAM - ᵉ; antismash - ᶠ; prokka - ᵍ; patric - ʰ

1 Discrepancies in gene identification across platforms are driven by variations in structural gene prediction mechanics (e.g., Prodigal vs. GLIMMER3), reference database composition, alignment algorithms (BLAST vs. Profile HMMs), stringency thresholds, and the specialized functional targets of the respective pipelines (e.g., primary vs. secondary metabolism)

### Pangenome comparative analysis and phylogenetic tree

**Fig. 3:**
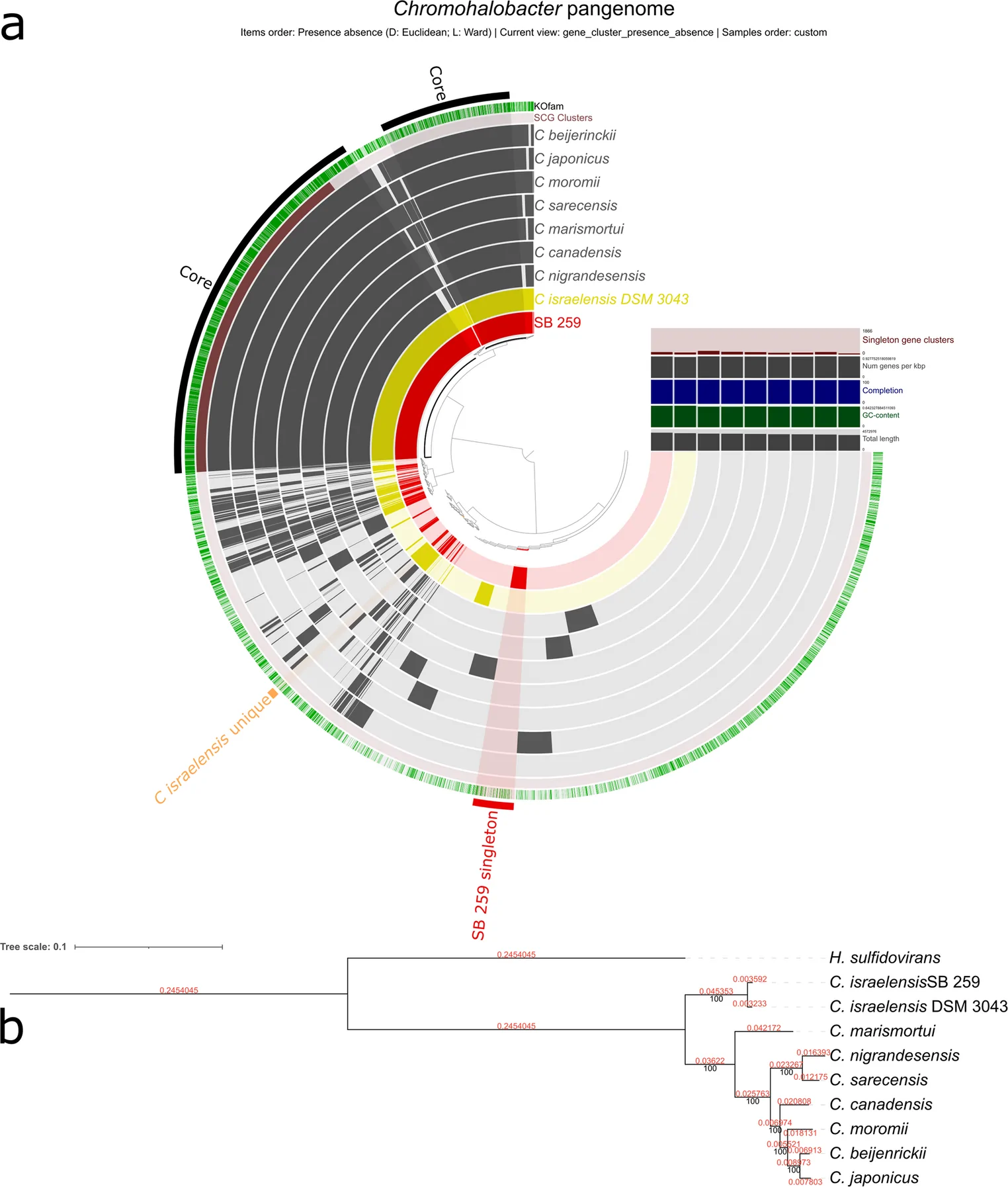
(**A**) Pangenome analysis graph. Highlighting the bins of singletons from SB259 (red), core genome (black) and genes shared by both *C. israelensis* genomes (orange). The SB 259 genome is colored red and the reference *C*. *israelensis* genome, yellow. The brown section indicates the Single Copy Gene clusters (SCGs), genes that appeared once in the genome. (**B**) Phylogenetic tree of SB 259 and the genus RefSeq genomes. Branch lengths are highlighted in red and bootstraps values in black^1^. *H. sulfidovirans* genome was used as the outgroup for rooting

The pangenome (Fig. 3A) revealed 2,223 gene clusters in the core genome (~ 70%), encompassing essential functions in sulfur cycling (sulfate reduction to sulfide) and nitrogen cycling (nitrate reduction to nitrite via the assimilatory pathway and ammonification via the dissimilatory pathway). These also included genes for amino acid biosynthesis (valine, leucine, lysine) and methane metabolism (incomplete pathways including: K00018, K00058, K00121, K00123, K00124, K00127, K00148, K00600, K00831, K00863, K01007, K01070, K01079, K01595, K01624, K01689, K01895, K11645, K15633, K21071, K22515) (Online Resource S3), collectively occupying 70% of the complete genome. Among the exclusive genes of the *C. israelensis* species (including the isolate from this study and the reference genome), 37 gene clusters were annotated. These included genes related to the synthesis of cysteine and methionine, as well as arginine and glutathione (Online Resource S4). A gene for starch and sucrose assimilation (K01087) was also annotated for both strains of the species, in addition to a gene for the transformation of sulfides into cysteine (K01738), linked to sulfur metabolism (Online Resource S4); in the other genomes of the genre the genes to sulfate reduction (K00380, K00381, K00390, K00955, K00956, K00957) were also present, but not the aforementioned. In the strain’s unique genome, 177 gene clusters were annotated (Online Resource S5). Among those with assigned functions, genes for purine metabolism (e.g., hpxW), genetic information processing (e.g., K03469), environmental information processing (e.g., cusF), and, primarily, amino acid metabolism were included. This specifically featured a gene for cysteine synthesis from sulfide and a gene for asparagine synthesis (asnB).

The phylogenetic tree exhibited distances consistent with the genus’s taxonomy, clustering the strain with the reference genome (Fig. 3B). Phylogenetic analysis revealed that the *C. israelensis* SB 259 lineage forms a strongly supported clade (bootstrap = 100) with *C. israelensis* DSM 3043, showing a low genetic distance (0.003592). This indicates high similarity between the two, corroborating the previously presented ANI and dDDH values that confirm both belong to the same species. It’s important to highlight the high phylogenetic proximity between these lineages, especially considering that although both are halophilic, they were isolated from distinct environments, suggesting broad ecological plasticity for the species. The clade formed by *C. israelensis* is close to *C. marismortui* (bootstrap = 100), yet maintains a considerable phylogenetic distance, as shown by the branch length (0.042172). The remaining lineages form two well-defined and distinct clades: one composed of *C. nigrandesensis* and *C. sarecensis*, and another by *C. canadensis*, *C. moromii*, *C. japonicus*, and *C. beijerinckii*, both exhibiting high internal cohesion.

## Discussion

This marks the first genome of *Chromohalobacter israelensis* obtained from marine sediments within a pockmark field [59–61] in the Santos Basin. Other genomes of this species have been assembled previously from samples of distinct marine environments, such as Caribbean Colombian sea sediment and tidal water from the coast of India [62, 63].

The genome of *C. israelensis* SB 259 is the largest within its genus (3.8 Mb) and is larger than other genomes of the species available in the NCBI database. The deep-sea marine environment from which this isolate was obtained can present stressful conditions for microorganisms, such as oligotrophy, low temperatures, and the absence of sunlight [64, 65], which could explain the presence of a large number of genes related to stress tolerance to conditions of such an environment.

Functional genomic analysis demonstrates the strain’s genomic potential to degrade and metabolize diverse substrates, ranging from sugars to more recalcitrant substances like lignin and cellulose, aligning with Arahal et al.‘s [15] description, a characteristic that may help to understand the broad range of habitats from which the species was isolated, since it could make the species more likely to obtain energy in diverse conditions. The functional annotation indicates that this microorganism have a putative metabolic capability to play roles in the sulfur cycle within the marine biosphere, if active in the environment, which would make it possible for it to contribute to sulfur cycling and to help maintain the balance and availability of sulfur compounds in the deep-sea environment, roles already highlighted for microorganisms in the deep Atlantic Ocean [66]. The classification by the MicroTrait software aligns with the iOA584 metabolic model previously reported [16], which classified 40% of enzymes as transport-related. The classification of 15.3% of genes into the amino acid subsystem by RAST annotation supports the reported capacity of the species to synthesize all 20 amino acids [16].

The annotation highlights the presence of genes related to stress tolerance, particularly resistance to osmotic stress. The annotated genes associated with the compatible solute ectoine constitute a complete biosynthesis cluster [67], in addition to the capacity for betaine osmoprotectant production [16]. The strain also exhibits the potential to survive in environments polluted by arsenic, thanks to the identified genes related to arsenic transformations, a capability previously reported for the genus in other environments [68, 69]. Additionally, the predicted functions of flagellum formation and chemotaxis are present in the genome, enabling the strain to detect and respond to environmental stimuli. The ectoine biosynthesis cluster represents a potential target for cloning methodologies and industrial production.

The pangenome justifies the assembled genome’s size by demonstrating the presence of unique genes relative to both the genus and the species itself. Representatives of this genus exhibit a highly diverse metabolism well-adapted to the conditions in which they are typically found. The unique genome of the strain in the present study underscores this finding by complementing an already diverse metabolism with additional pathways for, for example, amino acid synthesis. The presence of hpxW in the unique genome suggests a possible further adaptation to nutrient-limited conditions. However, not enough genes were found in the unique genome to suggest a selective pressure by the pockmark environment. The genus displayed a substantial core genome encompassing essential vital metabolic functions. Concurrently, the ANI analysis performed during the pangenome preparation indicated a value above the species identification threshold for *C. japonicus* and *C. beijerinckii*, highlighting the need for further investigation into the taxonomic identification of these species.

The high phylogenomic similarity with other *C. israelensis* strains suggests that the species is genetically quite conservative, despite the distinct environments from which they were collected [70, 71]. Furthermore, when compared to pangenomes of other genera within the same family [72–74], the core genome of the genus *Chromohalobacter* proved to be quite robust. Khaleque et al. [75] observed a selective pressure for the conservation of genes responsible for saline stress tolerance in the halophilic genus *Acidihalobacter*. A similar phenomenon can be suggested for the *Chromohalobacter* pangenome, which may also help to justify the diversity of environments where the genus has been sampled: Andean soils [76], hypersaline lake soils, solar salterns [70, 77, 78], marine sediment, and salt-preserved food products [79–81].

Long-read sequencing proved effective for obtaining the prokaryotic genome in this study, facilitating de novo assembly with optimal read lengths and high coverage. The strain was reliably identified as belonging to *Chromohalobacter israelensis*, a known halophile previously isolated from other hypersaline environments and serving as a model organism for osmoregulation studies. Based on the review conducted for this study, this is the first strain of *C. israelensis* to be isolated from pockmark fields globally, offering significant insights into the species’ evolution in diverse environments. Functional annotations revealed a metabolism potentially highly adapted for survival under various stresses, with a substantial portion of genes dedicated to resource acquisition and utilization, and stress resistance. This notably includes the ectoine synthesis cluster, a compatible solute of high industrial interest, capable of stabilizing cellular molecules without disrupting their functions. This cluster represents a potential target for the cloning and industrial production of ectoine.

In summary, the detailed analysis of the *C. israelensis* SB 259 genome, particularly through pangenome insights, underscores the species’ remarkable adaptability to extreme marine environments. The pangenome’s robust core highlights the conservation of essential metabolic and stress-response functions across the genus, aligning with observations in other halophilic taxa.

## Supplementary Information

Below is the link to the electronic supplementary material.

Supplementary Material 1

Supplementary Material 2

## Data Availability

The assembly generated can be found in the National Centre for Biotechnology Information (NCBI) database under BioProject PRJNA1270021.

## Abbreviations

HM: Halobacterium medium
ANI: Average nucleotide identity
dDDH: Digital DNA-DNA hybridization
MGEs: Mobile genetic elements
SCGs: Single copy genes
HGT: Horizontal gene transfer
TRAP: Tripartite ATP-independent periplasmic

## Footnotes

1
^The branch lengths represent the expected number of amino acid substitutions per site, calculated under the LG+Γ substitution model^.
